# Catastrophic expenditure and impoverishment of patients affected by 7 rare diseases in China

**DOI:** 10.1186/s13023-016-0454-7

**Published:** 2016-06-06

**Authors:** Xiao-Xiong Xin, Xiao-Dong Guan, Lu-Wen Shi

**Affiliations:** Department of Pharmacy Administration and Clinical Pharmacy, School of Pharmaceutical Sciences, Peking University, Beijing, 100191 China; International Research Center of Medicinal Administration, Peking University, Beijing, 100191 China

**Keywords:** Rare disease, Orphan drug, Catastrophic expenditure, Impoverishment effect, Affordability

## Abstract

**Background:**

China is actively promoting regulation of rare diseases, rare disease and orphan drugs have been formally incorporated into the national planning. However, few studies have been done to evaluate the affordability of rare disease patients in China. This study aims to provide policy recommendations for the establishment of social security mechanism for rare diseases in China, so as to address the problem of poverty caused by these diseases.

**Methods:**

A total of 7 rare diseases were selected by Delphi method. Affordability of treatment for the 7 rare diseases was assessed through annual per capital income, catastrophic expenditure and impoverishment expenditure among urban and rural residents in China.

**Results:**

Assessed through annual per capital income, health expenditure for the 7 rare diseases are all rather high. The highest health expenditure is equivalent to income of 69.34 years of one urban resident, and the burden is heavier for rural residents. Through catastrophic expenditure assessment, proportions of the population experiencing catastrophic expenditure caused by the 7 rare diseases are all under 0.167 ‰. However, once one is ill and taking medications, he will suffer from catastrophic health expenditure. Through impoverishment expenditure assessment, the proportions of impoverishment payment are low among both urban and rural residents, but the 7 rare diseases could lead nearly 4.6 million people into poverty on a national scale.

**Conclusion:**

The affordability of treatment for rare disease as well as orphan drugs is rather poor. Residents of different income levels all have difficulties to afford the treatment for rare diseases, so poverty caused by rare diseases is quite widespread. Therefore, social security mechanism for rare disease patients should be established and specific payment pattern for orphan drugs should be set up.

**Electronic supplementary material:**

The online version of this article (doi:10.1186/s13023-016-0454-7) contains supplementary material, which is available to authorized users.

## Background

Rare disease refers to the disease with low incidence which is hard to be diagnosed and treated [1]. Therapeutic drugs for rare diseases are often called orphan drugs. The standard of rare disease varies across countries [2]. Since 1980s, the legislation on rare diseases and polices towards orphan drugs have been gradually implemented in developed countries and regions [3].

Although China is actively promoting regulation of rare diseases, there is no legislation for rare disease up to now. Neither is registration system for rare diseases or official definition on rare disease [4]. In 2010, experts from Genetics Group of Chinese Medical Association put forward an unofficial standard for rare disease on Symposium on Definition of Rare Disease, which is genetic disease with prevalence rate lower than 1/500,000 in population or incidence rate lower than 1/10,000 in neonate. Current regulations only include incentive policies for registration and approval of orphan drugs. There are no detailed rules for development and pricing of orphan drugs. The nationwide reimbursement system for rare diseases has not been established [5]. With the rapid economic development, rare diseases are causing rising social concentration throughout the country. In January 2016, National Experts Committee of Diagnosis and Security of Rare Disease was set up under the leading of National Health and Family Planning Commission of People’s Republic of China. In February of the same year, Chinese Premier Li Keqiang proposed to promote the development and update of pharmaceutical industry at State Council executive meeting, emphasizing improving the industrial development of orphan drugs. It symbolised that rare disease and orphan drugs have been formally incorporated into the national planning.

The cost of diagnosis and treatment for rare disease is high, which may impoverish the patients and their families. Rare disease is not only a problem in health system but extends to social sphere. The design of legal system and policies of rare disease in China are far behind the need for pharmaceutical and social development. Therefore, it is of great social significance to explore the feasibility of establishing social security mechanism for rare diseases in China.

There have been some researches to investigate the medical insurance policies of rare diseases and orphan drugs in China, but few studies have been done to evaluate the affordability of rare disease patients in China [6]. Using Delphi method to select sampled rare diseases, our study assessed the affordability and providing suggestions of establishing social security mechanism for rare diseases.

## Methods

Our study evaluated the affordability of medicines by the following three methods. Firstly, WHO and Health Action International (HAI) have proposed a standard survey tool that applies the Lowest Paid unskilled Government Worker (LPGW) [7]. Affordability is then expressed in terms of the number of days the LPGW has to work to afford a course of treatment. Secondly, the catastrophic approach, i.e. the payment for a commodity is deemed catastrophic (unaffordable) when its cost exceed a certain percentage of household resources, was calculated [8]. Thirdly, according to the impoverishment approach, affordability of medicines was evaluated by measuring the proportion of the population that drops below the poverty line due to health expenditures of the diseases [9].

Due to the large gap between urban and rural areas in China, the affordability of rare diseases in this study intended to be assessed separately by urban and rural residents, and the affordability would be analysed by the above-mentioned three methods individually.

### Sampled diseases

Rare disease and therapeutic regimen needed to be identified to determine the health expenditure. This study used the Delphi method to conduct two rounds’ consultation of medical experts in Beijing. All of the ten experts were chief physicians from tertiary hospitals in Beijing, who specialised in department of neurology, cardiology, paediatrics, dermatology, respiratory, nephrology, ophthalmology and haematology and had rich experience on diagnosis and treatment of rare diseases. The selection criteria of diseases were to choose diseases with clear diagnosis standard and definite medical therapy. Then the selected diseases were categorized into three levels according to their health expenditure: health expenditure less than GDP per capita (level I), health expenditure between GDP per capita and three times of GDP per capita (level II), and health expenditure over three times of GDP per capita (level III). After the first round of consultation through email, 19 diseases were obtained (7 in level I, 6 in level II and 6 in level III). To further narrow down the disease scope, the experts were asked to select 2–3 diseases from each level. Eventually 7 rare diseases were selected which could be effectively treated with different health expenditures. After discussion of the experts, the standard medical therapy and annual health expenditure were determined. The prevalence data were retrieved from the European Orphanet database [10]. Because rare diseases usually require long-term treatment, this study used health expenditure per year as the measurement, as shown in Table 1.

**Table 1:** Sampled rare diseases and medicines

Rare disease	Prevalence	Therapeutic regimen	Health expenditure per year (1,000 yuan)	Health expenditures as a share of per capita GDP
Duchenne Muscular Dystrophy (DMD)	5/100000	Methylprednisolone Tablet, 0.6 mg/Kg/day	1.20	0–1
Alport Syndrome (AS)	2/100000	ACEI combined with ARB	7.50	0–1
Tuberous Sclerosis Complex (TSC)	8.8/100000	Sirolimus Tablet, 1 mg bid	40	0–1
Amyotrophic Lateral Sclerosis (ALS)	5/100000	Riluzole Tablet, 50 mg bid	55	1–3
Idiopathic Pulmonary Fibrosis (IPF)	16.7/100000	Pirfenidone Tablet, 200–400 mg tid	86	1–3
Pulmonary Arterial Hypertension (PAH)	5/100000	Bosentan Tablet, 125 mg bid	240	>3
Gaucher Disease (GD)	1/100000	Imiglucerase for Injection, 60 U/kg, fortnightly	2000	>3

### Per capital income approach

According to WHO/HAI standard survey tool, the affordability of medicines could assessed through annual per capital income [7]. We used this method to assess the affordability of medicines for rare diseases. Result is expressed in terms of the number of years, which measuring health expenditures with the share of annual per capital income. Health expenditures of sampled rare diseases of urban and rural residents were calculated separately.

### Catastrophic approach

**Fig. 1:**
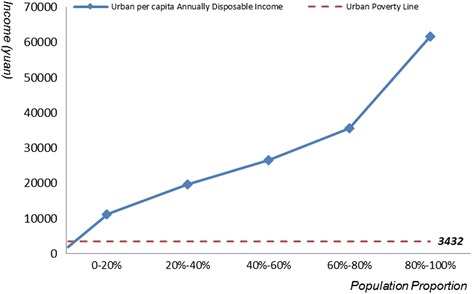
Distribution curve of Chinese urban per capita annually disposable income

**Fig. 2:**
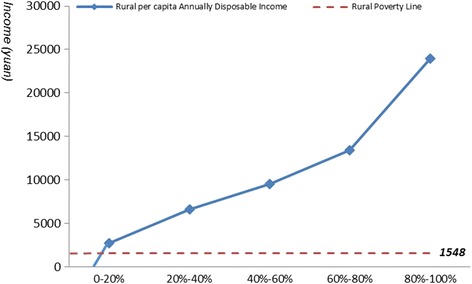
Distribution curve of Chinese rural per capita annually disposable income

WHO defined expenditure as catastrophic if a household’s health expenditure exceeded 40 % of remaining income after subsistence needs have been met [11]. We calculated the minimum annual income needed for avoiding catastrophe by this standard. We needed Chinese income distribution to calculate the catastrophe population and catastrophe rate. Income distribution curves of urban and rural residents were simulated separately by in this paper. We used annual disposable income per capita as coordinate, then divided the population into five groups and took the proportion of the population as the abscissa. The income of each group was hypothesized to have linear relation with the proportion of the population, as shown in the following equation:

$$ {y}_i={a}_i{x}_i+\mathrm{b}i $$

in which y was annual disposable income per capita and x was the proportion of the population. Distribution curves of annual per capita disposable income of urban and rural residents were shown in Figs. 1 and 2 [12]. According to the simulation curves, catastrophe rate caused by certain diseases could be calculated:

$$ \mathrm{Catastrophe}\ \mathrm{Rate}=\left(\mathrm{The}\ \mathrm{Minimum}\ \mathrm{Annual}\ \mathrm{Income}\ \mathrm{t}\mathrm{o}\ \mathrm{avoid}\ \mathrm{Catastrophe}-{\mathrm{b}}_i\right)/{a}_i\times 100\% $$

In addition, our study adjusted the threshold of catastrophic expenditure (30 %, 50 %) for sensitivity analysis.

### Impoverishment approach

The impoverishment approach investigates the extent to which expenditures on medicines push people’s income below a certain absolute threshold. The method aims to compare the proportion of the population below the poverty line before and after the procurement of medicines except those who had been already below the threshold before the procurement. This method needed to determine urban and rural poverty line at first, which is determined based on national rural and urban Minimum Living standard and graphed in Figs. 1 and 2. The most recent poverty line was issued in 2011, which was only applied for rural residents. Since rural and urban residents were considered as two groups in the study, the annual per capita subsidy for low-income residents in 2014 was taken as the poverty line for urban residents, which had already been done by other research [13]. Based on this standard, urban poverty line is 3432 yuan and rural poverty line is 1548 yuan. There are 18.77 million urban residents and 52.07 million rural residents [14].

Impoverishment Rate = amounts of impoverished population caused by disease/amounts of population not in poverty before disease × 100 %.

## Results

### Per capita income evaluation

In 2014, annual per capita disposable income of urban and rural residents is 28,843.9 yuan and 10,488.9 yuan respectively. Results are shown in Table 2.

**Table 2:** Affordability of the sampled rare diseases measured by per capita annually disposable income

Rare disease	Health expenditure per year (1,000 yuan)	Health expenditures as a share of per capita annually disposable income (year)	
		Urban	Rural
DMD	1.20	0.04	0.11
AS	7.50	0.26	0.72
TSC	40	1.39	3.81
ALS	55	1.91	5.24
IPF	86	2.98	8.20
PAH	240	8.32	22.88
GD	2000	69.34	190.68

i. Taking average annual income as reference, only the health expenditure of DMD and AS is lower than annual per capita income of urban and rural residents. That is to say, the health expenditure of rare disease is high, which generally means heavy burden on patients, especially to those rural residents with low income.
ii. The health expenditure of some rare diseases is terribly high. For example, the average annual health expenditure of GD is equivalent to 69.34 times of average annual income of urban residents and 32 times of high-income groups (top 20 %) of urban residents whose average annual disposable income are 61,615 yuan. For these rare diseases, the patients cannot afford the medical expenses for treatment.

### Catastrophic evaluation

According to the catastrophic method and income distribution curve, we calculated the population and proportion of urban or rural residents facing catastrophic expenditure owing to the treatment of 7 sampled rare diseases. Catastrophe and sensitive analysis results are shown in Table 3, taking the prevalence of disease into consideration.

**Table 3:** Catastrophic effect of expenditures and sensitivity analysis of sampled rare diseases

Rare disease	Threshold	The minimum annually income to avoid catastrophe	Catastrophe rate		Catastrophe population	
			Urban (‰)	Rural (‰)	Urban (1,000)	Rural (1,000)
DMD	30 %	5147	0.0021	0.0112	1.56	6.93
	40 %	3860	0.0015	0.0078	1.09	4.85
	50 %	3088	0.0011	0.0058	0.81	3.61
AS	30 %	32168	0.0125	0.0200	9.33	12.37
	40 %	24126	0.0086	0.0181	6.41	11.18
	50 %	19301	0.0058	0.0162	4.37	10.04
TSC	30 %	171563	0.0880	0.0880	65.93	54.44
	40 %	128673	0.0880	0.0880	65.93	54.44
	50 %	102938	0.0880	0.0880	65.93	54.44
ALS	30 %	235900	0.0500	0.0500	37.46	30.93
	40 %	176925	0.0500	0.0500	37.46	30.93
	50 %	141540	0.0500	0.0500	37.46	30.93
IPF	30 %	368861	0.1670	0.1670	125.11	103.32
	40 %	276646	0.1670	0.1670	125.11	103.32
	50 %	221317	0.1670	0.1670	125.11	103.32
PAH	30 %	1029380	0.0500	0.0500	37.46	30.93
	40 %	772035	0.0500	0.0500	37.46	30.93
	50 %	617628	0.0500	0.0500	37.46	30.93
GD	30 %	8578167	0.0100	0.0100	7.49	6.19
	40 %	6433626	0.0100	0.0100	7.49	6.19
	50 %	5146900	0.0100	0.0100	7.49	6.19

The study finds that, i. Taking 40 % as threshold, the catastrophe rate of sampled rare diseases does not exceed 0.1670‰ throughout the country, and the proportion of patients experiencing catastrophic expenditure with DMD is only 0.0015‰. However, taking the whole population into consideration, DMD will cause 6000 patients to experience catastrophic expenditure in China. There are about 7000 rare diseases known worldwide, but there is no statistical data in China. The rare disease patients who may incur catastrophic expenditure should be paid serious attention to.

ii. Except for DMD and ALS, the catastrophe rate of the other sampled rare diseases has no difference between urban and rural areas, which is equivalent to the prevalence of relevant rare diseases. It shows that these five diseases would bring huge burden to patients, who cannot afford the health expenditure. Once one is ill and taking medicines, he will suffer from catastrophic expenditure. Taken 40 % as threshold, a patient need an average annual income of over 6.4 million yuan to avoid catastrophic expenditure.

iii. Taken 40 % as threshold, DMD and AS have different influences on urban and rural residents. These two diseases cause 1.09 thousand and 6.41 thousand urban residents, 4.85 thousand and 11.18 thousand rural residents respectively to experience catastrophic expenditure.

iv. The sensitive analysis shows that the change of threshold leads no changes among sampled diseases except for the probability of catastrophic expenditure for patients with DMD and AS. It is indicated the stability of this evaluation method.

### Impoverishment evaluation

According to the impoverishment method, with the combination distribution curve and poverty line data, considering the prevalence of sampled rare diseases, proportion of impoverishment and number of impoverished patients are showed in Table 4.

**Table 4:** Impoverishing effect of sampled rare diseases

Rare disease	Urban		Rural	
	Impoverishment rate (‰)	Impoverishment population (1,000)	Impoverishment rate (‰)	Impoverishment population (1,000)
DMD	0.0006	0.43	0.0008	0.46
AS	0.0015	1.08	0.0084	4.76
TSC	0.0663	48.45	0.0880	49.86
ALS	0.0436	31.86	0.0500	28.33
IPF	0.1670	121.98	0.1670	94.62
PAH	0.0500	36.52	0.0500	28.33
GD	0.0100	7.30	0.0100	5.67

i. Impoverishment rate of sampled rare diseases is low among urban and rural residents. Among the 7 disease, impoverishment rate of IPF is the highest, up to 0.1670‰ both in urban and rural residents, while impoverishment rate of DMD in urban residents is the lowest (0.0006‰). However, the sampled rare diseases could lead 459.64 thousand people into poverty on a national scale. It shows that the problem of impoverishment by rare diseases in China is rather severe and should be paid more attention.
ii. Among the 7 diseases, the treatment burden of AS is relatively light and the impoverishment population of AS in rural residents is significantly more than that of urban residents, which indicate that policy makers should pay more attention to the disadvantaged groups when implementing policies. AS lead 1.08 thousand urban and 4.76 thousand rural residents into poverty.
iii. IPF leads more people to poverty than the other 6 rare diseases, which could impoverish 121.98 thousand urban and 94.62 thousand rural residents respectively.
iv. Impoverishment rate of IPF, PAH, GD in urban residents and that of TSC, ALS, IPF, PAH, GD in rural residents are equal to corresponding prevalence. This result indicates that once one is ill, he will fall into poverty for health expenditure.

## Discussion

Simoens found the price of orphan drugs is inversely proportional to the prevalence rate of the rare diseases treated [15]. Of the 7 surveyed diseases, however the aforesaid phenomenon was not observed. Our study showed that the prevalence rate of DMD, ALS and PAH were the same, while the health expenditure for the three diseases varied from 1200 to 240,000 yuan. The results also shown that poor households were less able to afford the health expenditure than richer households, which were consistent with other studies [16–18]. However, some rare diseases have higher incidence in low-income residents, such as leukemia in Gansu Province (higher in rural) and malaria in China [19, 20]. Therefore disease burden of rural residents should be paid more attention. Furthermore, the reality cannot fully be captured by the results of affordability assessment, because many patients would rather not use healthcare service than become impoverished [21, 22].

High price of orphan drugs is the direct cause of heavy burdens for rare diseases patients, which is consistent with other studies [23]. Therefore, there is an urgent need to cut down the price of these drugs. However, as the market scale is relatively small and most orphan drugs are monopolized, cutting down the prices alone still cannot make the drugs become affordable to many patients. It takes time to define rare disease and kick-start relevant legislation in China, but we think policy makes may as well enact reimbursement policy for rare disease patients to address the impoverishment of rare diseases. In this case, covering rare diseases in the health care system and paying the health expenditure by multiple sources may be an effective solution to ease the burden for patients with rare diseases. WHO suggests that in developing countries, the cost for prolonging a quality adjusted life year (QALY) is acceptable when it is less than 3 times of the average GDP per capita [24]. In 2014, China’s GDP per capita has reached 46,629 yuan [12], so the acceptable cost for each QALY can be as high as almost 140,000 yuan, making it practical to cover the rare diseases with effective treatment and moderate expenses in the health care system. Besides, China should fully negotiate and make use of social resources, encourage the NGOs and companies to donate and set up a payment pattern so that health care funds, patients and third parties can share the expenses in order to reduce the ratio of out-of-pocket (OOP) expenditure. In this way, more patients may probably afford proper medical care, and the problem of poverty caused by disease could also be solved.

The three methods applied in evaluating the affordability of rare diseases and orphan drugs all have certain limitations. The annual per capital income method uses residents’ annual income as indicator in calculation. However, the huge gap between the rich population and the poor makes average income an ineffective index to describe the affordability condition of the general public. With current data, we can specify the evaluation into five different groups by their income level, but this is still not satisfying in reflecting the conditions of some rich groups and the poor. As for catastrophic expenditure method, it uses the threshold 40 % defined by WHO standards. As treatment is usually more expensive for rare diseases, the threshold can be lifted accordingly. For calculation in both catastrophic expenditure and impoverishment expenditure method, the distribution curves of urban and rural annual per capita disposable income. When fitting the income curve, we assume that for each income group, the income is in linear distribution, which may lead to an overestimated affordability for some low income groups and an underestimated affordability for some high income groups [25].

Furthermore, there is no registration system for rare disease or nationwide epidemiological survey on rare disease in China, so the prevalence rate used in this study is from Orphanet. Most rare diseases are genetic diseases and characterized by district and race, which might cause biases for the catastrophe rate and impoverishment rate in China (e.g. if the prevalence rate is higher in China, catastrophe rate and impoverishment rate would be underestimated). Thus there is an urgent need to establish registration system for rare disease, collecting data like prevalence rate data and providing evidence for policy making and implement. The three methods share a common assumption in calculation that the patients need to cover the health expenditure all by themselves, neglecting the circumstances where expenditures could be co-paid by other sources, thus underestimating the actual affordability of patients. Some districts in China have covered certain rare diseases in the health care system. In Qingdao, a city in Shandong province, patients of PAH and GD can have part of the expenses reimbursed. In Shanghai, 12 kinds of rare diseases such as phenylketonuria, maple syrup urine disease, and tyrosinemia have been covered by the city medical insurance [26]. Patients could receive a maximum of 200,000 yuan per year as reimbursement for health expenditure.

## Conclusion

Our results shows that the affordability of treatment for rare disease is rather poor. Residents of different income levels all have difficulties to afford the treatment for rare diseases. Rare disease could easily impoverish patients and their families. Therefore, social security mechanism for rare disease patients should be established and specific payment pattern for orphan drugs should be set up. To reduce OOP health expenditure of patients, the co-payment pattern, led by government medical insurance and supported by medical aid, social charity and commercial insurance, is recommended.

## Abbreviations

WHO, World Health Organization; HAI, health action international; LPGW, lowest paid unskilled Government Worker; DMD, Duchenne muscular dystrophy; AS, Alport syndrome; TSC, tuberous sclerosis complex; ALS, amyotrophic lateral sclerosis; IPF, idiopathic pulmonary fibrosis; PAH, pulmonary arterial hypertension; GD, Gaucher disease.

## Additional file

Additional file 1: Catastrophic expenditure and impoverishment of patients affected by 7 rare diseases in China. **Table S1.** Annual Disposable Income and Linear Equation for Each Group of Urban Population. **Table S2.** Annual Disposable Income and Linear Equation for Each Group of Rural Population. (DOC 42 kb)
