# Supplementary material for: Catastrophic expenditure and impoverishment of patients affected by 7 rare diseases in China
Source: Orphanet J Rare Dis. 2016 Jun 6;11:74. doi: 10.1186/s13023-016-0454-7 (PMC4895890; doi:10.1186/s13023-016-0454-7)
Supplement: Additional file 1: — Catastrophic expenditure and impoverishment of patients affected by 7 rare diseases in China. Table S1. Annual Disposable Income and Linear Equation for Each Group of Urban Population. Table S2. Annual Disposable Income and Linear Equation for Each Group of Rural Population. (DOC 42 kb) [file 13023_2016_454_MOESM1_ESM.doc]

**Additional File - Catastrophic expenditure and impoverishment of patients affected by 7 rare diseases in China**

Table S1 Annual Disposable Income and Linear Equation for Each Group of Urban Population

| Urban Cumulative Population Proportion  (%) | Urban Per Capita Average Annual Disposable Income (yuan) | Linear Equation |
| --- | --- | --- |
| 0-20 | 11219.3 | Y_1_ = 103906X_1_ + 828.65 |
| 20-40 | 19650.5 | Y_2_ = 42156X_2_ + 7003.7 |
| 40-60 | 26650.6 | Y_3_ = 35001X_3_ + 9150.4 |
| 60-80 | 35631.2 | Y_4_ = 44903X_4_ + 4199.1 |
| 80-100 | 61615.0 | Y_5_ = 129919X_5_ - 55312 |
| * urban poverty line = 3432 yuan, while the urban poverty population proportion = 2.51%  **average remaining income after subsistence needs/average total income = 77.72% | | |

Table S2 Annual Disposable Income and Linear Equation for Each Group of Rural Population

| Rural Cumulative Population Proportion  (%) | Rural Per Capita Average Annual Disposable Income (yuan) | Linear Equation |
| --- | --- | --- |
| 0-20 | 2768.1 | Y_1_ = 77070X_1_ - 4938.9 |
| 20-40 | 6604.4 | Y_2_ = 19182X_2_ + 849.95 |
| 40-60 | 9503.9 | Y_3_ = 14498X_3_ + 2255.1 |
| 60-80 | 13449.2 | Y_4_ = 19727X_4_ - 359.35 |
| 80-100 | 23947.4 | Y_5_ = 52491X_5_ - 23295 |
| * rural poverty line = 1548 yuan, while the rural poverty population proportion = 8.42%  **average remaining income after subsistence needs/average total income = 77.72% | | |
